# Racial, ethnic and sex disparity in acute heart failure patients with COVID-19: A nationwide analysis

**DOI:** 10.1016/j.heliyon.2024.e34513

**Published:** 2024-07-20

**Authors:** Anas Hashem, Amani Khalouf, Mohamed Salah Mohamed, Tarek Nayfeh, Ahmed Elkhapery, Salman Zahid, Ahmed Altibi, Harshith Thyagaturu, Anthony Kashou, Nandan S. Anavekar, Martha Gulati, Sudarshan Balla

**Affiliations:** a Department of Internal Medicine, Rochester General Hospital, Rochester, NY, USA; b Department of Cardiovascular Medicine, Allegheny General Hospital, Pittsburg, PA, USA; c Evidence-based Medicine, Mayo Clinic School of Medicine, Rochester, MN, USA; d Department of Cardiovascular Medicine, Oregon Health & Science University, Portland, OR, USA; e Electrophysiology & Cardiac Arrhythmia Program, Department of Cardiovascular Medicine, Yale-New Haven Hospital, New Haven, CT, USA; f Department of Cardiovascular Medicine, West Virginia University, Morgantown, WV, USA; g Department of Cardiovascular Medicine, Mayo Clinic School of Medicine, Rochester, MN, USA; h Department of Preventive Cardiology, Barbra Streisand Women's Heart Center, Cedars Siani, Los Angeles, CA, USA

**Keywords:** COVID-19, Acute heart failure, Racial disparity, In-hospital mortality, Clinical outcomes

## Abstract

**Background:**

Patients with acute heart failure (AHF) exacerbation are susceptible to complications in the setting of COVID-19 infection. Data regarding the racial/ethnic and sex disparities in patients with AHF and COVID-19 remains limited.

**Objective:**

We aim to evaluate the impact of race, ethnicity, and sex on the in-hospital outcomes of AHF with COVID-19 infection using the data from the National Inpatient Sample (NIS).

**Methods:**

We extracted data from the NIS (2020) by using ICD-10-CM to identify all hospitalizations with a diagnosis of AHF and COVID-19 in the year 2020. The associations between sex, race/ethnicity, and outcomes were examined using a multivariable logistic regression model.

**Results:**

We identified a total of 158,530 weighted AHF hospitalizations with COVID-19 infection in 2020. The majority were White (63.9 %), 23.3 % were Black race, and 12.8 % were of Hispanic ethnicity, mostly males (n = 84,870 [53.5 %]). After adjustment, the odds of in-hospital mortality were lowest in White females (aOR 0.83, [0.78–0.98]) and highest in Hispanic males (aOR 1.27 [1.13–1.42]) compared with White males. Overall, the odds of cardiac arrest (aOR 1.54 [1.27–1.85]) and AKI (aOR 1.36 [1.26–1.47] were higher, while odds for procedural interventions such as PCI (aOR 0.23 [0.10–0.55]), and placement on a ventilator (aOR 0.85 [0.75–0.97]) were lower among Black males in comparison to White males.

**Conclusion:**

Male sex was associated with a higher risk of in-hospital mortality in white and black racial groups, while no such association was noted in the Hispanic group. Hispanic males had the highest odds of death compared with White males.

## Abbreviations and acronyms

**AHF**: Acute heart failure
**AHRQ**: Agency for Healthcare Research and Quali**ty**
**AKI**: acute kidney injury
**AMI**: acute
myocardial infarction
**aOR**: adjusted odds ratio
**CABG**: coronary artery bypass graft
**CAD**: coronary artery disease
**CCR**: cost-to-charge ratio
**CKD**: chronic kidney disease
**COH**: costs of hospitalization
**COPD**: chronic obstructive pulmonary disease
**COVID-19**: The Coronavirus disease 2019
**CV**: Cardiovascular
**CVA**: cerebrovascular accident
**ECMO**: extracorporeal membrane oxygenation
**ESRD**: end-stage renal disease
**HCUP**: Healthcare Cost and Utilization Project
**IABP**: intra-aortic balloon pump
**ICD-10-CM**: International Classification of Diseases, 10th Revision, and Clinical Modification
**ICU**: Intensive care unit
**IQR**: interquartile range
**LOS**
Length of stay
**LVAD**: Left ventricular assist device
**MCS**: mechanical cardiac support
**MI**: myocardial infarction
**NIS**: National Inpatient Samplze
**PCI**: percutaneous coronary artery intervention
**pVAD**: percutaneous ventricular-assisted device
**PVD**: peripheral vascular disease
**US**: United States

## 1 Introduction

Cardiovascular (CV) disease is the leading cause of death in the United States (US) [1]. Despite favorable trends in age-adjusted CV disease-related mortality in the US over the last 2 decades, the outcomes in minorities, who comprise up to 40 % of the US population, have lagged behind [2,3]. Several studies have shown that sex, race, and ethnicity impact clinical outcomes even after adjusting for baseline comorbidities [[4], [5], [6], [7]]. Specifically, healthcare access, delivery, and insurance coverage vary among patients based on their sex, race and ethnicity in the US [[8], [9], [10]]. Structural racism has also been identified as a critical reason for the persistence of such health disparities in the US [11]. The Coronavirus disease 2019 (COVID-19) pandemic has also demonstrated racial and ethnic disparities regarding clinical outcomes [[12], [13], [14], [15], [16]]. Males and minorities were predominantly affected during the pandemic. With high mortality due to COVID-19, minorities contributed up to 36 % of COVID-19 deaths in the US. Furthermore, Cronin et al. reported excess mortality in Black non-Hispanic males during the pandemic year 2020 from non-COVID related illness compared with individuals from other races and ethnicities [17]. Also, studies have revealed significantly higher rates of intensive care unit (ICU) mortality among Black race and Hispanic ethnicity individuals with COVID-19 compared to White race individuals [[18], [19], [20]]. Muhyieddeen et al. utilized a population database to evaluate the effect of COVID-19 pandemic on racial and ethnic minorities among pateints with acute myocardial infarction and their analysis revealed a significant racial/ethnic disparity being worse among Black race patients and Hispanic ethnicities [21].

Acute heart failure (AHF) is a serious medical condition that impacts individuals regardless of their sex, race or ethnicity. Moreover, studies have found that concomitant COVID-19 infection with AHF is associated with worse in-hospital mortality and health outcomes [[22], [23], [24]]. It is mostly unknown if the COVID-19 pandemic had an impact on the existent racial, ethnic, and sex-based disparities previously reported in heart failure literature. Therefore, we sought to assess outcomes amongst patients with AHF and concurrent COVID-19 infection stratified by race, ethnicity, and sex utilizing a contemporary database of inpatient hospitalizations.

## 2 Methodology

### 2.1 Data Source and ethical consideration

The National Inpatient Sample (NIS) is one of several databases managed by the Agency for Healthcare Research and Quality (AHRQ) through a Federal-State-Industry partnership called the Healthcare Cost and Utilization Project (HCUP). The NIS 2020 database contains administrative claims data from more than 7 million inpatient hospitalizations annually in 43 participating states plus the District of Columbia, representing more than 97 % of the USA population. To calculate hospitalization cost, which includes total expenses incurred to provide services, cost-to-charge ratio (CCR) files were used. The CCR files provide hospital-specific ratios to calculate hospitalization costs based on the specific hospitalization characteristics. Institutional Review Board approval and informed consent were not required for this study because NIS data are de-identified and publicly available. As per HCUP guidelines, observations with a cell count 10 or less are reported as "<11".

### 2.2 Study sample and patient selection

The NIS sample design encompasses the hospital universe, comprising all hospitals operational at any point during the calendar year, designated as community hospitals by the American Hospital Association (AHA) [25]. This framework consists of a stratified sample drawn from a subset of hospitals in states contributing data to the HCUP project. With 60 distinct strata, hospitals are stratified based on region, location/teaching status (within the region), bed size category (within region and location/teaching status), and ownership (within region, location/teaching, and bed size categories). The delineation of regions aligns with the four census regions (Northeast, Midwest, South, and West), while location is categorized as urban or rural according to AHA's classification. Bed size categories are defined as small, medium, and large, with unique size thresholds established for each combination of hospital region, teaching status, and urban/rural designation. Within each stratum, a systematic random sampling approach is employed to select hospitals, with the sample size set at 20 percent of the universe for that stratum. Hospitals are arranged based on the first three digits of their zip codes for systematic sampling. Discharge sample weights are then computed within each sampling stratum, representing the ratio of discharges in the universe to those in the sample [25,26].

We analyzed NIS data using the International Classification of Diseases, 10th Revision, and Clinical Modification (ICD-10-CM) claims codes. ICD-10-CM codes I5021, I5023, I5031, I5033, I5041, and I5043 were used to identify patients hospitalized with AHF exacerbation (primary diagnosis). To identify COVID-19 cases, we used the ICD-10 code of U071 (secondary diagnoses). We used weighted hospitalization sample for our study as weighted the data provides a nationally representative findigns [27]. All diagnosis and procedure fields were queried to select and categorize the study population; ICD-10 codes are reported in Supplementary Table 1. Individuals under 18 years old and those who were admitted electively were excluded. Missing information regarding race, ethnicity, death status, and races other than White and Black and ethnicities other than hispanic were also excluded from the study. Despite being different, the terms “race” and “ethnicity” have used interchangeably in medical literature [28]. According to AHRQ, “The five race categories are now Black or African American, White, Asian, American Indian or Alaska Native, and Native Hawaiian or Other Pacific Islander”, while Hispanic is reported as an ethnicity [29]. However in our study, we reported a comparison of clinical outcomes between specific races and Hispanic ethnicity including Black race individuals, White race individuals, and Hispanic individuals as other individuals from other races and ethnicities had small sample size, failing to meet stastical power to detect difference in clinical outcomes. A detailed flowchart of derivation of the study cohort is presented in Supplemental Fig. 1.

### 2.3 Variables

Baseline patient characteristics included demographic variables (age, sex, race, ethnicity, median house income, and primary payer), hospital characteristics (teaching status and bed size), and discharge disposition were obtained. We also collected the baseline comorbidities, which included hypertension, diabetes mellitus, dyslipidemia, hypothyroidism, obesity, smoking, chronic obstructive pulmonary disease (COPD), chronic kidney disease (CKD), end-stage renal disease (ESRD), peripheral vascular disease (PVD), prior myocardial infarction (MI), prior percutaneous coronary artery intervention (PCI), coronary artery disease (CAD), coronary artery bypass graft (CABG), cerebrovascular accident (CVA), and atrial fibrillation. Charlson's comorbidity index was calculated and divided into 3 categories (1, 2, and ≥3). A detailed explanation of all variables in the NIS is available online [26].

### 2.4 Study outcomes

The primary study endpoint was in-hospital mortality. Secondary endpoints included 1) Clinical outcomes such as, acute myocardial infarction (AMI), cardiogenic shock, cardiac arrest, bleeding, stroke, acute kidney injury (AKI), and respiratory failure; 2) Rates of procedural interventions including PCI, CABG, mechanical cardiac support (MCS), intra-aortic balloon pump (IABP), percutaneous ventricular-assisted device (VAD), extracorporeal membrane oxygenation (ECMO), Left ventricular assist device (LVAD), vasopressors, need for dialysis, and ventilator; 3) resource utilization was studied using length of stay (LOS), costs of hospitalization (COH) and palliative care consults, and 4) the distribution of AHF with COVID-19 hospitalizations and mortality rate across the USA using USA Divisions (as defined by HCUP) based on the patients’ race and ethnicity.

### 2.5 Statistical analysis

Data were stratified by race, ethnicity, and sex. To compare groups in terms of baseline characteristics and outcomes, categorical data were presented as counts and percentages and compared using Pearson's Chi-square test. Continuous data median and interquartile range (IQR) and comparisons between sexes in the same race and ethnicity were conducted using the non-parametric Mann-Whitney *U* test, while comparisons between all race-ethnicity-sex groups were performed following the Kruskal Wallis test. A multivariate logistic regression model was utilized to compare in-hospital mortality between the different groups using White male as a reference, and adjusting for age, Charlson comorbidity index, hospital bed size, teaching status, and insurance. The results of the logistic regression analysis were presented as adjusted odds ratios (aOR) with 95 % confidence intervals. To calculate representative estimates and standard errors, our analysis accommodated for the complex sampling design of the NIS database including the different strata, hospital clusters and weights provided in the NIS sample [26,30,31]. As per the HCUP recommendations, we utilized heirarchial regression in building our models, and hospital clusters and strata were accounted for in the model as random intercepts and slopes [25]. For all analyses, a two-tailed p-value of ≤0.05 was considered statistically significant. We used Microsoft Excel® to display the USA map distribution (based on USA Divisions) [32] of AHF with COVID-19 hospitalizations and mortality rates classified based on the patients' race and ethnicity. All statistical analyses were performed using R version 4.2.2 and SPSS version 26 (IBM Corp, Armonk, NY).

## 3 RESULTS

### 3.1 Baseline characteristics

A total of 158,530 weighted hospitalizations of AHF with COVID-19 infection were identified in 2020. The majority were White (63.9 %), 23.3 % were Black race, and 12.8 % were of Hispanic ethnicity. Mostly were males (n = 84,870 [53.5 %]) and they were generally younger than females across all racial and ethnic groups (W: 77 years vs. 78 years, p < 0.01; B: 66 y vs. 70 y, p < 0.01; H: 67 y vs. 72 y, p < 0.01). Hypertension, obesity, hypothyroidism, and COPD were more common in females, whereas cardiac comorbidities (including prior MI, PCI, CAD, CABG, and atrial fibrillation), smoking, and ESRD were higher in males. Hypertension was highest among Black females (93.3 %). Prior history of CAD was more prevelant among White males (55.4 %). White race patients had higher Charlson index scores (≥3) compared with Black race patients. Further details regarding baseline comorbidity and hospital characteristics of the study population are shown in Table 1, Table 2, respectively.

**Table 1:** Baseline characteristics of the study population based on their race, ethnicity and sex.

Variable n (%)	White Race			Black Race			Hispanic Ethnicity			Group p-value
	Male (n = 55,085 [54.4 %])	Female (n = 46,225 [45.6 %])	p-value	Male (n = 18,125 [49.0 %])	Female (n = 18,850 [51.0 %])	p-value	Male (n = 11,660 [57.6 %])	Female (n = 8585 [42.4 %])	p-value	
Age (Median, [IQR])	77 [68–83]	78 [71–86]	**<0.01**	66 [56–74]	70 [60–79]	**<0.01**	67 [57–77]	72 [62–82]	**<0.01**	**<0.01**
**Comorbidities**										
Hypertension	88.1 %	89.1 %	**<0.01**	93.0 %	93.3 %	0.22	87.7 %	91.1 %	**<0.01**	**<0.01**
Diabetes Mellitus	30.2 %	27.1 %	**<0.01**	33.8 %	36.0 %	**<0.01**	39.4 %	42.5 %	**<0.01**	**<0.01**
Dyslipidemia	58.3 %	54.4 %	**<0.01**	50.6 %	51.0 %	0.49	53.1 %	55.2 %	**<0.01**	**<0.01**
Hypothyroidism	14.2 %	29.4 %	**<0.01**	4.9 %	11.4 %	**<0.01**	9.8 %	21.5 %	**<0.01**	**<0.01**
Obesity	24.9 %	28.7 %	**<0.01**	27.6 %	39.0 %	**<0.01**	24.3 %	30.8 %	**<0.01**	**<0.01**
Smoking	32.7 %	23.9 %	**<0.01**	24.2 %	19.7 %	**<0.01**	24.3 %	14.5 %	**<0.01**	**<0.01**
COPD	35.4 %	38.4 %	**<0.01**	27.5 %	29.7 %	**<0.01**	16.6 %	20.4 %	**<0.01**	**<0.01**
CKD	40.9 %	37.8 %	**<0.01**	39.5 %	37.9 %	**<0.01**	30.6 %	30.7 %	0.91	**<0.01**
ESRD	6.6 %	5.5 %	**<0.01**	19.7 %	17.8 %	**<0.01**	20.6 %	18.6 %	**<0.01**	**<0.01**
PVD	2.2 %	2.0 %	**<0.01**	1.4 %	1.9 %	**<0.01**	2.8 %	2.7 %	0.83	**<0.01**
Prior MI	14.5 %	10.1 %	**<0.01**	10.3 %	9.3 %	**<0.01**	12.6 %	8.6 %	**<0.01**	**<0.01**
Prior PCI	14.7 %	9.0 %	**<0.01**	8.7 %	7.1 %	**<0.01**	11.6 %	7.0 %	**<0.01**	**<0.01**
History of CAD	55.4 %	38.9 %	**<0.01**	38.5 %	32.8 %	**<0.01**	45.0 %	35.8 %	**<0.01**	**<0.01**
History CABG	16.4 %	6.7 %	**<0.01**	6.1 %	4.6 %	**<0.01**	11.5 %	6.8 %	**<0.01**	**<0.01**
Prior stroke/TIA	5.5 %	5.8 %	0.06	7.9 %	8.4 %	0.06	5.4 %	5.1 %	0.38	**<0.01**
Atrial fibrillation	34.2 %	32.8 %	**<0.01**	22.9 %	20.5 %	**<0.01**	22.3 %	21.5 %	0.23	**<0.01**
**Charlson Comorbidity Index**										
1	11.6 %	16.6 %	**<0.01**	16.9 %	17.3 %	0.11	20.3 %	20.8 %	0.10	**<0.01**
2	31.8 %	35.4 %		35.9 %	36.5 %		36.1 %	37.1 %		
≥3	56.7 %	47.9 %		47.2 %	46.2 %		43.6 %	42.1 %		
Nursing home/facility	29.4 %	37.4 %		26.5 %	27.1 %		18.5 %	21.4 %		
Home Health Care	17.3 %	18.7 %		13.5 %	21.2 %		15.1 %	18.3 %		

CABG: coronary artery bypass graft; CAD: Coronary Artery Disease; CKD: chronic kidney disease; COPD: Chronic obstructive pulmonary disease; CVA: cerebrovascular accident; DM: Diabetes Mellitus; ESRD: end-stage renal disease; PVD: peripheral vascular disease.

*As per HCUP regulations, observations with cell count <11 are reported as “<11”

**Table 2:** Hospital and insurance characteristics of the study population based on their race, ethnicity and sex.

Variable n (%)	White Race			Black Race			Hispanic Ethnicity			Group p-value
	Male (n = 55,085 [54.4 %])	Female (n = 46,225 [45.6 %])	p-value	Male (n = 18,125 [49.0 %])	Female (n = 18,850 [51.0 %])	p-value	Male (n = 11,660 [57.6 %])	Female (n = 8585 [42.4 %])	p-value	
**Hospital Teaching Status**										
Rural	11.2 %	11.3 %	0.87	6.5 %	6.3 %	0.53	1.8 %	1.5 %	**<0.01**	**<0.01**
Urban nonteaching	18.9 %	18.8 %		12.1 %	11.8 %		19.3 %	20.8 %		
Urban teaching	69.9 %	69.9 %		81.5 %	81.9 %		78.9 %	77.8 %		
**Hospital Bed size**										
Small	23.2 %	24.4 %	**<0.01**	20.7 %	20.5 %	0.21	19.4 %	20.6 %	0.07	**<0.01**
Medium	28.5 %	28.7 %		27.0 %	27.8 %		28.1 %	28.1 %		
Large	48.3 %	47.0 %		52.3 %	51.7 %		52.5 %	51.3 %		
**Median Household Income in the Zip code**										
0–25th percentile	26.7 %	25.8 %	**<0.01**	54.5 %	55.9 %	0.05	41.2 %	42.6 %	**0.02**	**<0.01**
26th-50th percentile	30.1 %	30.1 %		22.6 %	21.8 %		26.5 %	26.0 %		
51st-75th percentile	25.1 %	25.0 %		14.0 %	13.8 %		21.0 %	21.4 %		
76th-100th percentile	18.2 %	19.1 %		8.8 %	8.5 %		11.3 %	10.0 %		
**Primary Payer**										
Medicare	78.2 %	85.8 %	**<0.01**	61.9 %	71.7 %	**<0.01**	59.1 %	65.3 %	**<0.01**	**<0.01**
Medicaid	4.6 %	3.9 %		14.6 %	12.6 %		19.6 %	20.0 %		
Private insurance	12.5 %	8.5 %		16.2 %	13.6 %		14.3 %	9.6 %		
Self-pay/other	1.0 %	0.7 %		2.7 %	1.1 %		3.3 %	3.1 %		
**Disposition**										
Home	26.6 %	20.5 %	**<0.01**	35.5 %	30.0 %	**<0.01**	37.4 %	33.1 %	**<0.01**	**<0.01**
Nursing home/facility	29.4 %	37.4 %		26.5 %	27.1 %		18.5 %	21.4 %		
Home Health Care	17.3 %	18.7 %		13.5 %	21.2 %		15.1 %	18.3 %		

*As per HCUP regulations, observations with cell count <11 are reported as “<11”

### 3.2 Clinical outcomes

#### 3.2.1 In-hospital mortality

**Fig. 1:**
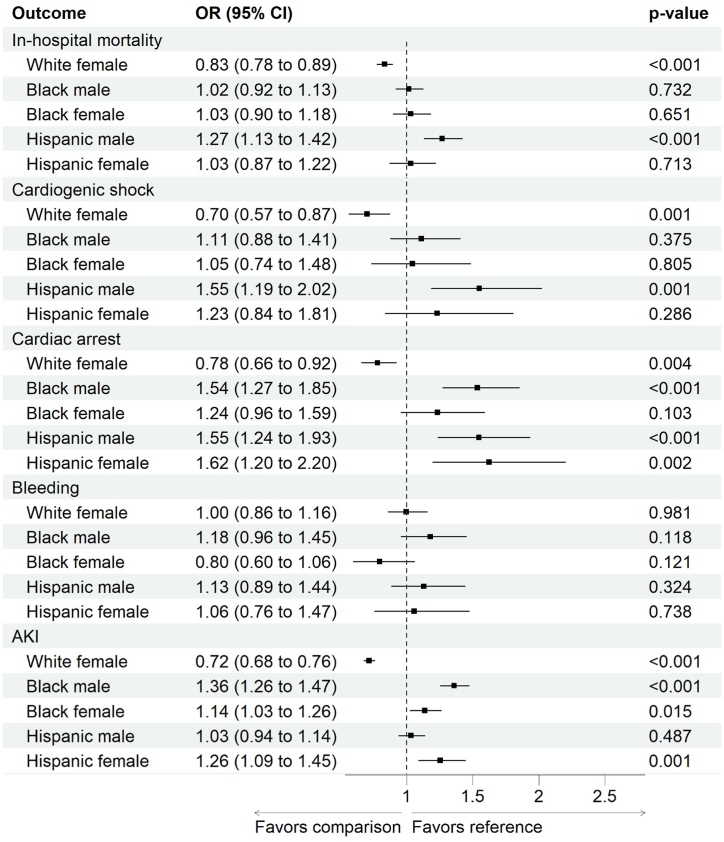
Adjusted odds ratio (aOR) for in-hospital mortality and complications classified based on the race, ethnicity and sex. OR was adjusted for Charlson comorbidity index, hospital bed size, hospital teaching status, USA divisions, and insurance using white male as a reference.

For crude in-hospital mortality of AHF and COVID-19 infection, those with Hispanic ethnicity had the highest mortality rates (24.6 %) followed by Non-Hispanic White individuals (22.1 %), and Non-Hispanic Black race individuals (19.4 %) (p < 0.01). The highest mortality rate was among Hispanic males (25.0 %), whereas the lowest was in Black females (19.0 %). The mortality rate was higher among males compared with females in both White (23.1 % vs. 20.8 %, p < 0.01) and Black (19.9 % vs. 19.0 %, p = 0.03) populations, with no differences by sex in the Hispanic ethnicity population (25.0 % vs. 24.1 %, p = 0.11). After adjustment for age, Charlson comorbidity index, hospital bed size, hospital teaching status, and insurance, using White males as the reference, White females had significantly lower odds of in-hospital mortality (OR = 0.83, 0.78–0.89 [95 % CI], p < 0.01), while Hispanic males had higher odds of in-hospital mortality (OR = 1.27, 1.13–1.42 [95 % CI], p < 0.01), Fig. 1.

#### 3.2.2 Clinical complications

The crude incidence rate of acute MI, cardiogenic shock, stroke, and respiratory failure was generally higher among males with an acute heart failure exacerbation hospitalization in all the racial and ethnic groups. Only cardiac arrest was more common in Hispanic females (7.2 % vs. 5.9 %, p < 0.01). The most common in-hospital complication was AKI which was also higher among White males compared with White females (46.9 % vs. 39.4 %, p < 0.01) and Black males compared with Black females (52.5 % vs. 48.6 %, p < 0.01) with no sex difference among the Hispanic population (45.8 % vs. 44.6 %, p = 0.08). Details on all the secondary outcomes are reported in Table 3. On multivariable analysis, Black male patients had higher odds of AKI (aOR 1.41, 95%CI [1.30–1.53]), and cardiac arrest (aOR 1.53, 95%CI [1.27–1.84]), with no differences in in-hospital mortality (aOR 1.02, 95%CI [0.92–1.13]) compared with White male. The adjusted complication outcomes are presented in Fig. 1.

**Table 3:** Unadjusted outcomes of acute heart failure and COVID-19 stratified by race, ethnicity and sex.

Variable n (%)	White Race			Black Race			Hispanic Ethnicity			Between Races p-value^a^
	Male (n = 55,085 [54.4 %])	Female (n = 46,225 [45.6 %])	p-value	Male (n = 18,125 [49.0 %])	Female (n = 18,850 [51.0 %])	p-value	Male (n = 11,660 [57.6 %])	Female (n = 8585 [42.4 %])	p-value	
**Clinical Outcomes**										
In-hospital Mortality	23.1 %	20.8 %	**<0.01**	19.9 %	19.0 %	**0.03**	25.0 %	24.1 %	0.11	**<0.01**
Acute myocardial infarction	5.7 %	4.0 %	**<0.01**	5.0 %	3.8 %	**<0.01**	8.6 %	7.5 %	**<0.01**	**<0.01**
Cardiogenic shock	2.1 %	1.3 %	**<0.01**	3.1 %	2.1 %	**<0.01**	4.2 %	3.3 %	**<0.01**	**<0.01**
Cardiac arrest	3.4 %	2.6 %	**<0.01**	5.6 %	5.3 %	0.17	5.9 %	7.2 %	**<0.01**	**<0.01**
Bleeding	3.8 %	3.8 %	0.74	4.3 %	3.5 %	**<0.01**	4.2 %	4.5 %	0.26	**<0.01**
Stroke	2.4 %	1.8 %	**<0.01**	3.1 %	2.7 %	**0.02**	3.5 %	2.7 %	**<0.01**	**<0.01**
AKI	46.9 %	39.4 %	**<0.01**	52.5 %	48.6 %	**<0.01**	45.8 %	44.6 %	0.08	**<0.01**
Palliative care consult	16.8 %	17.6 %	**<0.01**	10.0 %	12.1 %	**<0.01**	12.5 %	14.2 %	**<0.01**	**<0.01**
**Procedural Outcomes**										
PCI	0.6 %	0.4 %	**<0.01**	0.2 %	0.2 %	0.64	0.6 %	0.8 %	0.33	**<0.01**
CABG	0.1 %	0.0 %	**<0.01**	0.1 %	0.0 %	**<0.01**	0.3 %	0.1 %	**<0.01**	**<0.01**
Circulatory support	0.4 %	0.2 %	**<0.01**	0.3 %	0.2 %	**<0.01**	0.7 %	0.5 %	**<0.01**	**<0.01**
ECMO	0.1 %	NR	**<0.01**	0.2 %	0.3 %	0.07	0.4 %	0.2 %	**0.02**	**<0.01**
Vasopressors	3.7 %	2.9 %	**<0.01**	5.6 %	5.5 %	0.73	6.3 %	6.0 %	0.44	**<0.01**
Need for dialysis	4.3 %	3.7 %	**<0.01**	13.2 %	12.3 %	**<0.01**	14.7 %	12.8 %	**<0.01**	**<0.01**
Need for ventilator	12.2 %	10.9 %	**<0.01**	11.5 %	11.3 %	0.54	11.4 %	12.2 %	0.08	0.36
**Resource Utilization- All Cases**										
Length of stay	7 [4–12]	7 [4–11]	**0.03**	7 [4–14]	7 [4–14]	0.19	7 [4–15]	7 [4–13]	0.15	0.18
Total charges	56,670 [30,431–112,619]	53,135 [29,372–99,518]	**<0.01**	62,923 [32,418–135,262]	60,727 [32,384–125,187]	0.36	90,413 [42,435–200,725]	84,218 [41,686–180,818]	0.12	**<0.01**
**Resource Utilization- Surviving Cases**	42,385 (53.6 %)	36,630 (46.4)	**p-value**	14,510 (48.7)	15,260 (51.3)	**p-value**	8740 (57.3)	6520 (42.7)	**p-value**	**Between Races p-value**^a^
Length of stay	6 [4–11]	6 [4–11]	0.67	6 [4–12]	7 [4–12]	**0.04**	6 [3–13]	7 [4–12]	0.18	**<0.01**
Total charges	50,720 [28,359–94,365]	48,846 [28,071–85,643]	**<0.01**	53,838 [29,182–108,489]	53,984 [29,847–103,620]	0.86	68,976 [35,456–141,551]	68,358 [37,702–137,444]	0.74	**<0.01**

AKI: Acute kidney injury; PCI: Percutaneous coronary intervention; CABG: Coronary artery bypass graft; ECMO: extracorporeal membrane oxygenation.

Bleeding: defined as GI bleeding, bleeding during a procedure, and need for blood transfusion.

Circulatory support includes intra-aortic balloon pump, mechanical cardiac support and percutaneous ventricular assist device.

NR: not reported percentages for cell count <11 as per HCUP requirements.

a Race p-value is the p-value for chi square for categorical variable comparison.

#### 3.2.3 Procedural interventions

Regarding procedural interventions, mechanical ventilation was the most common intervention. Among White patients, mechanical ventilation was higher in males compared to females (12.2 % vs. 10.9 %, p < 0.01). but there was no sex differences amongst other races or ethnicity. Regardless of race and ethnicity, males had significantly higher rates of dialysis compared to females, with Hispanic males having the highest rate of dialysis (14.7 % vs. 12.8 %, p < 0.01), followed by Black males (13.2 % vs. 12.3 %, p < 0.01), and White males (4.3 % vs. 3.7 %, p < 0.01). Data on additional procedures during the COVID-19 era are shown in Table 3. On multivariable analysis, Black males were less likely to undergo PCI (aOR 0.23, 95%CI [0.10–0.55]), percutaneous VAD (aOR 0.21, 95%CI [0.07–0.60]) or to be placed on a ventilator (aOR 0.85, 95%CI [0.75–0.97]) compared with White males. White females were less likely to undergo circulatory support (aOR 0.53, 95 %CI [0.30–0.92]), require vasopressors (aOR 0.80, 95 %CI [0.68–0.94]), and placement on ventilator (aOR 0.89, 95 %CI [0.81–0.97]) in comparison with White males. A summary of the adjusted procedural intervention is shown in Supplemental Fig. 2.

#### 3.2.4 Resources utilization

Overall, Hispanic patients had a higher median hospitalization cost (M: $90,413 and F: $84,218, p = 0.12) followed by Black (M: $62,923 and F: $60,727, p = 0.36), and White (M: $56,670 and F: $53,135, p < 0.01) patients. Also, Hispanic patients had significantly higher costs compared with Black (p < 0.01) and White (p < 0.01) patients, Supplemental Figs. 3A and 3B. The LOS was longer in White males compared with White females (7 [4–12] vs. 7 [4–11] days, p = 0.11), with no sex differences seen in other races or ethnicities. Black and Hispanic patients had longer LOS compared with White patients (p < 0.01), Supplemental Figs. 3C and 3D. Palliative care consults were more common among females regardless of their racial and ethnic group compared with men (White: 17.6 % vs. 16.8 %, p < 0.01; Black: 12.1 % vs. 10.0 %, p < 0.01; Hispanic: 14.2 % vs. 12.5 %, p < 0.01).

For the resource utilizations of the surviving patients, we excluded dead patients from the analysis. Regarding the length of stay, there was no difference between White and Hispanic males and females, but black females had a higher length of stay compared with black males (p = 0.04). For the total hospital charges, there were no gender differences except between white males and females with higher costs among white males (p < 0.01), Table 3.

#### 3.2.5 Geographic trends in hospitalization and mortality

The racial distribution of hospitalized patients with both AHF and COVID-19 varied across the US. The East North Central had the highest percentage of hospitalized White patients at 23.3 %. For Black patients, the highest rates of hospitalizations in Black patients were seen in the South Atlantic (30.3 %), and Hispanic patients were more frequently hospitalized in the Pacific (27.3 %).

**Fig. 2:**
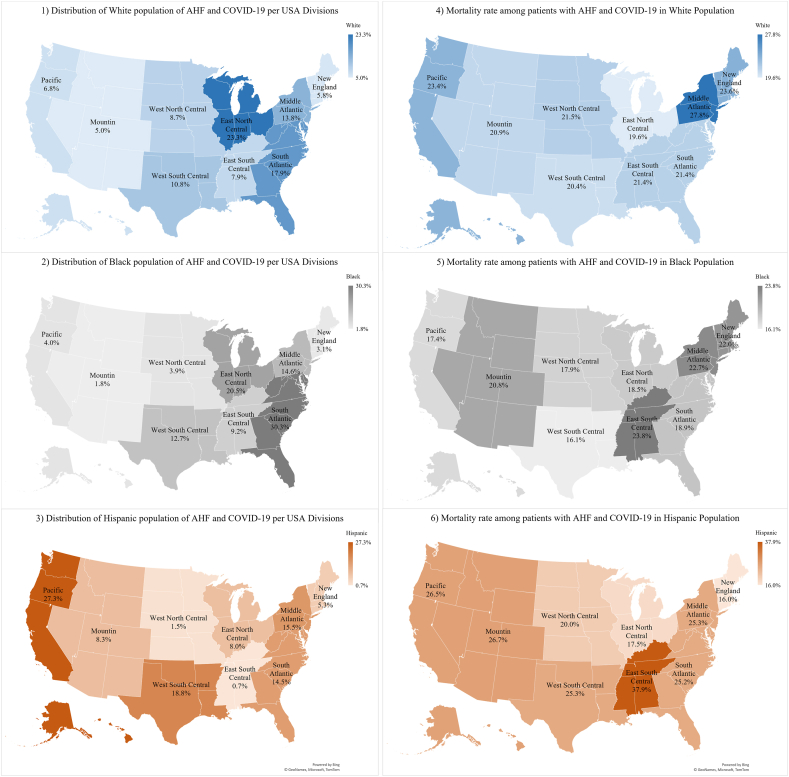
Distribution of AHF with COVID-19 based on race, ethnicity (1–3), and mortality rate per race, ethnicity/state (4–6).

The mortality rate among hospitalized patients also varied geographically. White patients had the highest mortality rates in the Middle Atlantic (27.8 %), and Black patients had the highest mortality rates in the East South Central (23.8 %), Middle Atlantic (22.7 %), and New England (22.0 %) regions. Hispanic patients had the highest mortality rates in the East South Central (37.9 %) region. Fig. 2 provides visual insight into the racial and geographic patterns of AHF and COVID-19 outcomes in the US. Males and females with AHF and COVID-19 infections were mostly distributed in East North Central (M: 20.7 %; F: 20.7 %), and South Atlantic (M:19.9 %; F: 20.9 %). Supplementary Fig. 4 visualizes the graphical distribution of sex with AHF and COVID-19 in the US.

**Fig. 3:**
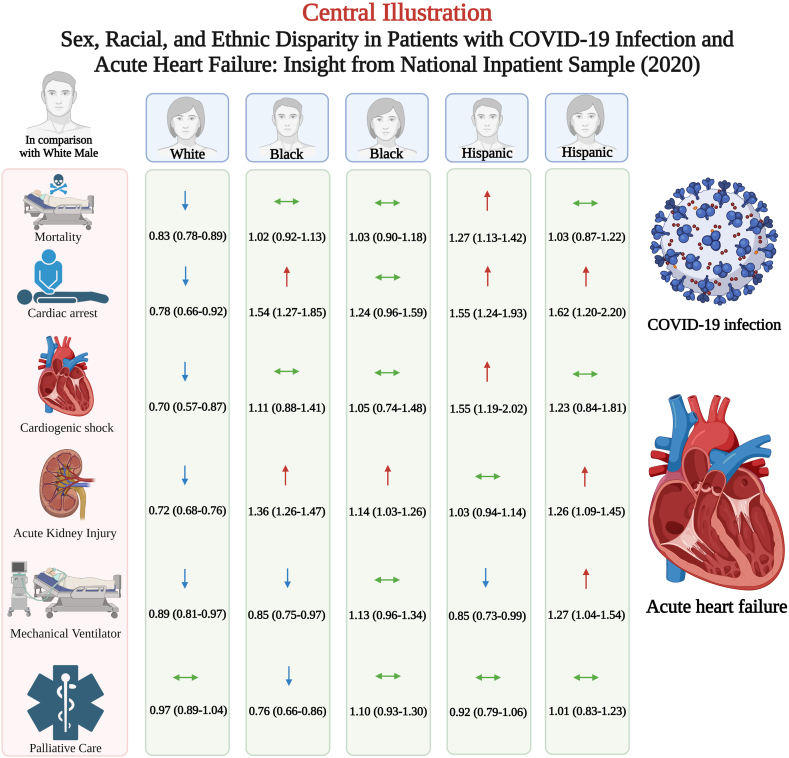
Central illustration.

Central illustration shown in Fig. 3 summarizes the study outcomes.

## 4 Discussion

In this study, we analyzed a nationwide database to evaluate for sex, racial, and ethnic disparities in the clinical outcomes of patients diagnosed with AHF and COVID-19 infection, which yielded the following noteworthy findings: 1) The in-hospital mortality rate was overall higher in males compared with females, 2) Odds of in-hospital mortality in those with AHF were highest for Hispanic males and lowest for White females, 3) Black patients of either sex had similar risk of mortality as White males, 4) Blacks and Hispanics had high rates of use of dialysis when presenting with AHF and COVID-19; and 5) There were higher rates of stroke and cardiogenic shock in Black and Hispanic individuals.

Members of minor racial and ethnic groups carry a disproportionate burden of heart failure with higher prevalence and 30-day age-adjusted mortality rate when compared to White race individuals [33]. Similarly, hospitalization rates and readmission rates for AHF are higher in Black race individuals. Studies have ascribed this disparity to inequities in access to care, pharmacotherapy, and advanced therapies for heart failure [34]. Our study suggests that the COVID-19 pandemic compounded the existing disparities in heart failure outcomes, with the minority population who were more adversely affected by COVID-19. We found that Hispanic individuals had the highest mortality rate, and the mortality risk persisted even after adjusting for baseline characteristics, including their socio-economic status. Male sex has already been identified as an independent factor associated with higher rates of in-hospital mortality due to COVID-19, similar to the finding in our study [[35], [36], [37], [38]]. Studies have reported a higher incidence of COVID-19, COVID-19-related hospitalizations, and mortality in underrepresented racial and ethnic groups [34]. A meta-analysis by Magesh et al. of 4 million patients reported minorities such as Hispanics and Blacks had higher rates of COVID-19 infection, ICU admissions, and unfavorable disease severity leading to worse clinical outcomes and higher death rates [14]. Another study by Ricardo et al. found that Hispanic patients have higher odds of death compared with non-hispanic White patients, with almost a similar aOR found in our study (1.44 [95%CI, 1.12–1.84]) [39]. Furthermore, the National Center for Health Statistics data showed a large relative increase in deaths of Hispanic ethnicity and Black populations compared to White populations due to heart and cerebrovascular diseases during the COVID-19 pandemic [40].

It is noteworthy to mention that there was no increased risk of mortality in Black including both males and females population compared with White males in both sexes after adjustment. This is in contrast to other studies that have reported worse outcomes in this racial group [14,40]. Our findings are consistent with the study by Rodriguez et al. who reported outcomes in 7868 patients with COVID-19 infection. The study showed no differences in in-hospital mortality, or major adverse cardiovascular events between White, Black or Hispanic [41]. Prior work has demonstrated that Black persons have a higher incidence of out-of-hospital sudden cardiac arrest in comparison with Whites in an analysis using emergency medical service systems data in the US, which may explain the lack of difference in mortality by race [[42], [43], [44], [45]]. Additionaly, Black patients were less likely to be diagnosed with COVID-19 infection in comparison with White patients in the US [46], and our analysis was only in those who were diagnosed with the COVID-19 infection.

Traditionally, the disparities in heart failure have been attributed to differences in cardiovascular comorbidity burden [47]. However, social determinants of health (SDOH) play a significant role in determining health care outcomes [48]. Multiple studies have suggested association of SDOH with adverse cardiovascular outcomes [[49], [50], [51], [52], [53], [54]]. SDOH are deemed to have played a vital role for the disparities noted during the COVID-19 pandemic. The majority of Black and Hispanic patients had presence of socioeconomic determinants such as belonging to a neighborhood in the lowest quartile for median household income and having significantly higher rates of Medicaid or self-pay, while lower rates of Medicare as the primary payer compared with White patients. Other studies have shown a similar low rate of healthcare coverage for minorities [55]. Access to healthcare during the pandemic was also an issue, especially during the lockdown, and also patients’ fear of contracting COVID-19 may have resulted in delays in seeking care. Further compounding the racial and ethnic disparity, especially during the COVID-19 pandemic, is the lack of trust in healthcare by minorities and misinformation pertaining to COVID-19 [56]. In addition, explicit and implicit bias in the healthcare infrastructure could have led to poor healthcare delivery to marginalized groups. For example, Yong et al. reported that Asians and Blacks were less likely to have undergone cardiovascular procedures during the early pandemic phase [57,58].

### 4.1 Disparity mortality and clinical outcomes

In terms of clinical complications and outcomes, we found that Hispanic ethnicity individuals had the highest rates of acute MI, cardiogenic shock, cardiac arrest, bleeding, and respiratory failure. Black males were less likely to undergo PCI, Black males and females were less likely to undergo pVAD and vasopressor use compared to White males as a reference. Few studies have reported Hispanic ethnicity and Black race population to have worse in-hospital clinical outcomes and lower rates of procedural interventions during the early months of the pandemic [14,59,60]. In addition, White females were less likely to undergo mechanical circulatory support, placed on vasopressors or ventilator with no difference in PCI intervention compared with White males. Similar findings were shown in a study published by Mehta et al. [61].

## 5 Strengths and limitations

Our study represents a large-scale, population-based analysis of patients who were hospitalized with AHF in the US in the year of 2020. It provides an estimate of hospitalization trends across the initial year of COVID-19, including clinical outcomes, procedural interventions, and utilization of hospital resources, which may aid in establishing or changing the current health care policies. However, there are several limitations in our study including, the inherent shortcomings of the NIS database as it is based on administrative claims for billing purposes using the ICD codes that is subjected to coding errors (selection bias), although specificity likely remains high [62]. The use of a single ICD-10 code (U071) for COVID-19 infection was not useful in determining the severity of COVID-19 infection (whether asymptomatic, symptomatic, upper respiratory tract infection or COVID-19 pneumonia). Meanwhile, according to Bhatt et al., the use of U071 as a code was specific across the year 2020 reaching up to 99.9 % with a modest sensitivity due to the lack of initial awareness and familiarity with this ICD-10 coding for COVID-19 [63]. Racial and ethnic demographics are self-described, making it subjectively identified. Therefore, the chances of erroneous allocations of patients to wrong race and ethnicity categories cannot be ruled out [64]. Other races (e.g. Asians, Native Americans, etc) were not described in out analysis. Additionally, NIS does not capture all the social determinants of health, such as language, education, housing insecurity, and food insecurity. Also the claims of any reported diagnoses (e.g., AHF, COPD, CVA, etc.) or complications (e.g., acute MI, septic shock, acute PE, etc.) are not supported by any laboratory, hemodynamic, imaging or echocardiographic data. Lastly, deaths outside the hospital are not reported in the NIS database, which limits extrapolation beyond hospitalizations.

## 6 Conclusions

Our study revealed the presence of significant racial and ethnic and sex disparities in outcomes of patients with COVID-19 infection and AHF. Targeted efforts should be made to address the unique needs of vulnerable populations, with access to care being of utmost importance at all times regardless of their race, ethnicity or sex.

## Data availability statement

Data included in article/supplementary material/referenced in article.

## Ethics declarations

Review and/or approval by an ethics committee was not needed for this study because NIS data are de-identified and publicly available. As per HCUP ethical guidelines, observations with a cell count 10 or less are reported as "<11".

## CRediT authorship contribution statement

**Anas Hashem:** Writing – original draft, Validation, Methodology, Formal analysis, Conceptualization. **Amani Khalouf:** Writing – original draft, Resources, Methodology, Investigation, Formal analysis, Conceptualization. **Mohamed Salah Mohamed:** Software, Methodology, Investigation, Formal analysis, Data curation, Conceptualization. **Tarek Nayfeh:** Visualization, Validation, Software, Conceptualization. **Ahmed Elkhapery:** Visualization, Validation, Supervision, Formal analysis, Data curation, Conceptualization. **Salman Zahid:** Software, Data curation, Conceptualization. **Ahmed Altibi:** Resources, Data curation, Conceptualization. **Harshith Thyagaturu:** Writing – review & editing, Visualization, Validation, Resources, Data curation, Conceptualization. **Anthony Kashou:** Writing – review & editing, Resources, Data curation, Conceptualization. **Nandan S. Anavekar:** Writing – review & editing, Visualization, Investigation, Data curation, Conceptualization. **Martha Gulati:** Writing – review & editing, Visualization, Validation, Supervision. **Sudarshan Balla:** Writing – review & editing, Validation, Supervision, Software, Resources.

## Declaration of competing interest

The authors declare that they have no known competing financial interests or personal relationships that could have appeared to influence the work reported in this paper.
