# Supplementary material for: Racial, ethnic and sex disparity in acute heart failure patients with COVID-19: A nationwide analysis
Source: Heliyon. 2024 Jul 20;10(15):e34513. doi: 10.1016/j.heliyon.2024.e34513 (PMC11327804; doi:10.1016/j.heliyon.2024.e34513)

SUPPLEMENTARY

# Supplementary Table 1: ICD-10 diagnosis at baseline and outcomes, and procedural codes used for the study

| Variables | ICD-10 |
| --- | --- |
| Acute heart failure (both reduced and preserved ejection fraction) | I50.21, I50.23, I50.31, I50.33, I50.41, and I50.43 |
| COVID-19 infection | U07.1 |
| Diabetes Mellitus | E10.0, E10.1, E10.6, E10.8, E10.9, E11.0, E11.1, E11.6, E11.8, E11.9, E12.0, E12.1, E12.6, E12.8, E12.9, E13.0, E13.1, E13.6, E13.8, E13.9 |
| Hypertension | I10.x, O10.x |
| Coronary Artery Disease | I251x, I255, I257x, I258x, I259x |
| Prior MI | I252 |
| Prior PCI | Z955, Z9861 |
| Prior CABG | I2571, I25708, I25709, Z951, I25812, I25810, I2579, I2576, I2573, I2572, I2572, I25700 |
| Atrial fibrillation | I480, I4891, I482, I481 |
| End stage heart failure | I5084 |
| PVD | I70.x |
| CVA | G45.x, G46.x, H34.0, I65.x–I69.x |
| COPD | I27.8, I27.9, J40.x–J47.x, J60.x–J67.x, J68.4, J70.1, J70.3 |
| Pulmonary circulation disorder | I27.x, I28.0, I28.8, I28.9 |
| CKD | N18.x |
| ESRD | Z99.2, N18.6 |
| Obesity | E66.x |
| Acute myocardial infarction | I2101, I2102, I2109, I2111, I212, I2121, I2129, I220, I221, I228, I229, I214, I222 |
| Percutaneous coronary intervention | 02703x, 02713x, 02733x |
| Need for pressors | 3E030XZ, 3E033XZ, 3E040XZ, 3E043XZ, 3E050XZ, 3E053XZ, 3E060XZ, 3E063XZ |
| VA-ECMO | 5A15223, 5A1522F, 5A1522G, 5A1522H, 5A15A2F, 5A15A2G, 5A15A2H, 5A0522C, 5A0512C, 5A1522G |
| Impella | 02HA3RZ |
| IABP | 5A02210 |
| Cardiogenic shock | R570 |
| Cardiac arrest | I462, I468, I469 |
| Hematoma | L7631, L7632, L7633, L7634 |
| Bleeding during a procedure | L7601, L7602, L7611, L7612 |
| Need for blood transfusion | 30230H0, 30230H1, 30230N0, 30230N1, 30230P0, 30230P1, 30230Q0, 30230Q1, 30230R0, 30230R1, 30233H0, 30233H1, 30233N0, 30233N1, 30233P0, 30233P1, 30233Q0, 30233Q1, 30233R0, 30233R1, 30240H0, 30240H1, 30240N0, 30240N1, 30240P0, 30240P1, 30240Q0, 30240Q1, 30240R0, 30240R1, 30243H0, 30243H1, 30243N0, 30243N1, 30243P0, 30243P1, 30243Q0, 30243Q1, 30243R0, 30243R1 |
| Hemorrhagic stroke | I6000, I6001, I6002, I6010, I6011, I6012, I602, I6020, I6021, I6022, I6030, I6031, I6032, I604, I6050, I6051, I6052, I606, I607, I608, I609, I610, I611, I612, I613, I614, I615, I616, I618, I619, I6200, I6201, I6202, I6203, I621, I629 |
| Ischemic stroke | I6300, I63011, I63012, I63013, I63019, I6302, I63031, I63032, I63033, I63039, I6309, I6310, I63111, I63112, I63113, I63119, I6312, I63131, I63132, I63133, I63139, I6319, I6320, I63211, I63212, I63213, I63219, I6322, I63231, I63232, I63233, I63239, I6329, I6330, I63311, I63312, I63313, I63319, I63321, I63322, I63323, I63329, I63331, I63332, I63333, I63339, I63341, I63342, I63343, I63349, I6339, I6340, I63411, I63412, I63413, I63419, I63421, I63422, I63423, I63429, I63431, I63432, I63433, I63439, I63441, I63442, I63443, I63449, I6349, I6350, I63511, I63512, I63513, I63519, I63521, I63522, I63523, I63529, I63531, I63532, I63533, I63539, I63541, I63542, I63543, I63549, I6359, I636, I638, I6381, I6389, I639 |
| Need for a ventilator | 5A09357, 5A09358, 5A09359, 5A0935A, 5A0935B, 5A0935Z, 5A09457, 5A09458, 5A09459, 5A0945A, 5A0945B, 5A0945Z, 5A09557, 5A09558, 5A09559, 5A0955A, 5A0955B, 5A0955Z |
| Acute kidney injury | N170, N171, N172, N178, N179 |

**Supplemental Figure 1:** Study Flow Chart

Nationwide Inpatient Sample (NIS) database for all hospitalization in 2020 using ICD-10 Diagnostic Codes of I5021, I5023, I5031, I5033, I5041, and I5043 to identify patients who were diagnosed with acute heart failure.

Excluded (n=542,790)

- Patients less than 18 years of age (n=6,590)
- Elective admission (n=296,250)
- Missing race (n=70,435)
- Missing death status (n=860)
- Other races (n=169,515)

Included cases (n=3,233,789)

COVID-19 (n=158,530)

Without COVID-19 (n=3,075,259)

Eligibility

Weighted acute heart failure hospitalizations (n=3,777,439)

Grouping

Analysis

Hispanic (n=20,245)

White (n=101,310)

Black (n=36,975)


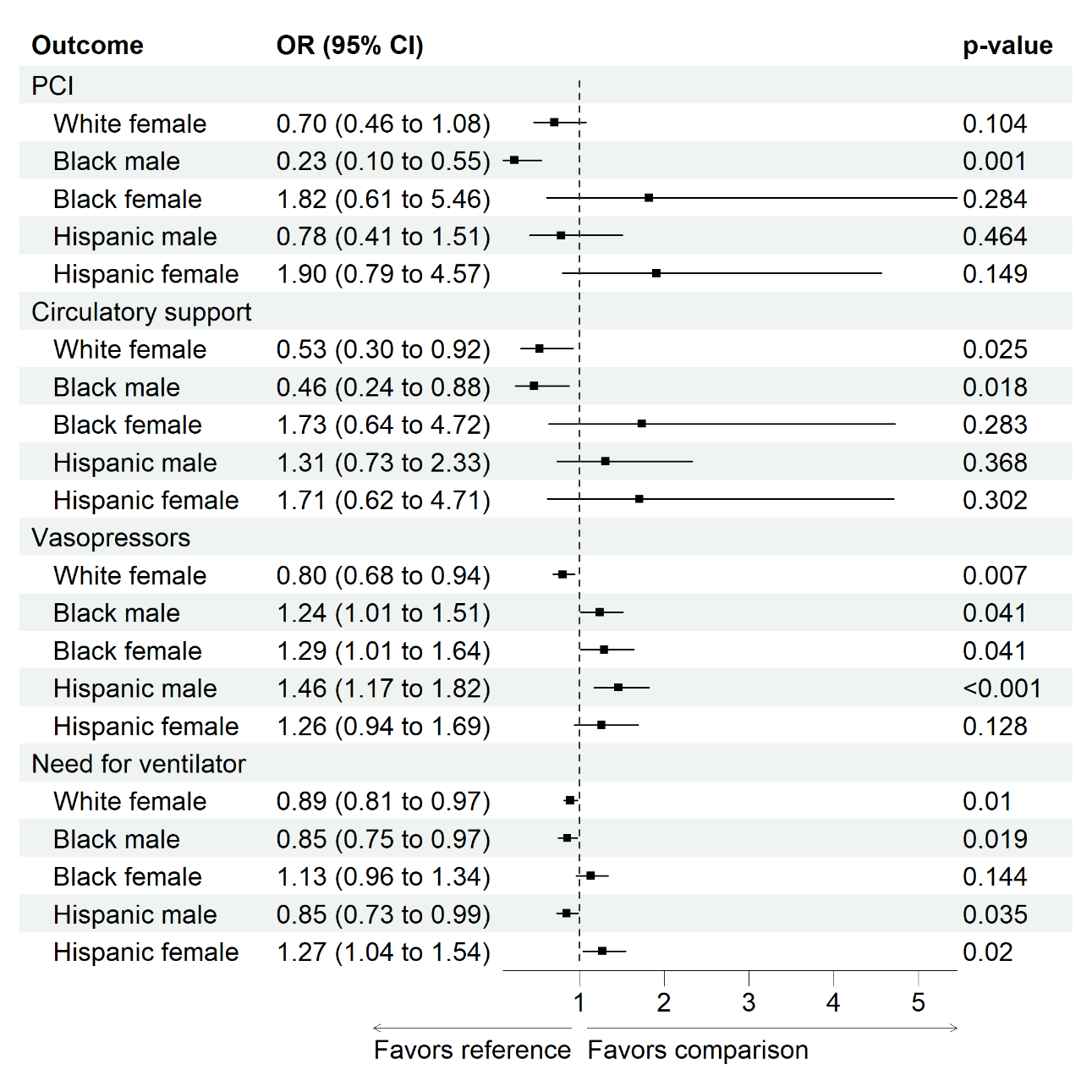
**Supplemental Figure 2:** Adjusted odds ratio (aOR) for procedural outcomes classified based on the race and sex. OR was adjusted for Charlson comorbidity index, hospital bed size, hospital teaching status, USA divisions and insurance using white male as a reference.

**Supplementary** **Figure 3:** Resource utilization including costs of hospitalization based on sex (3A) and race (3B), and length of stay based on sex (3C) and race (3D).

**
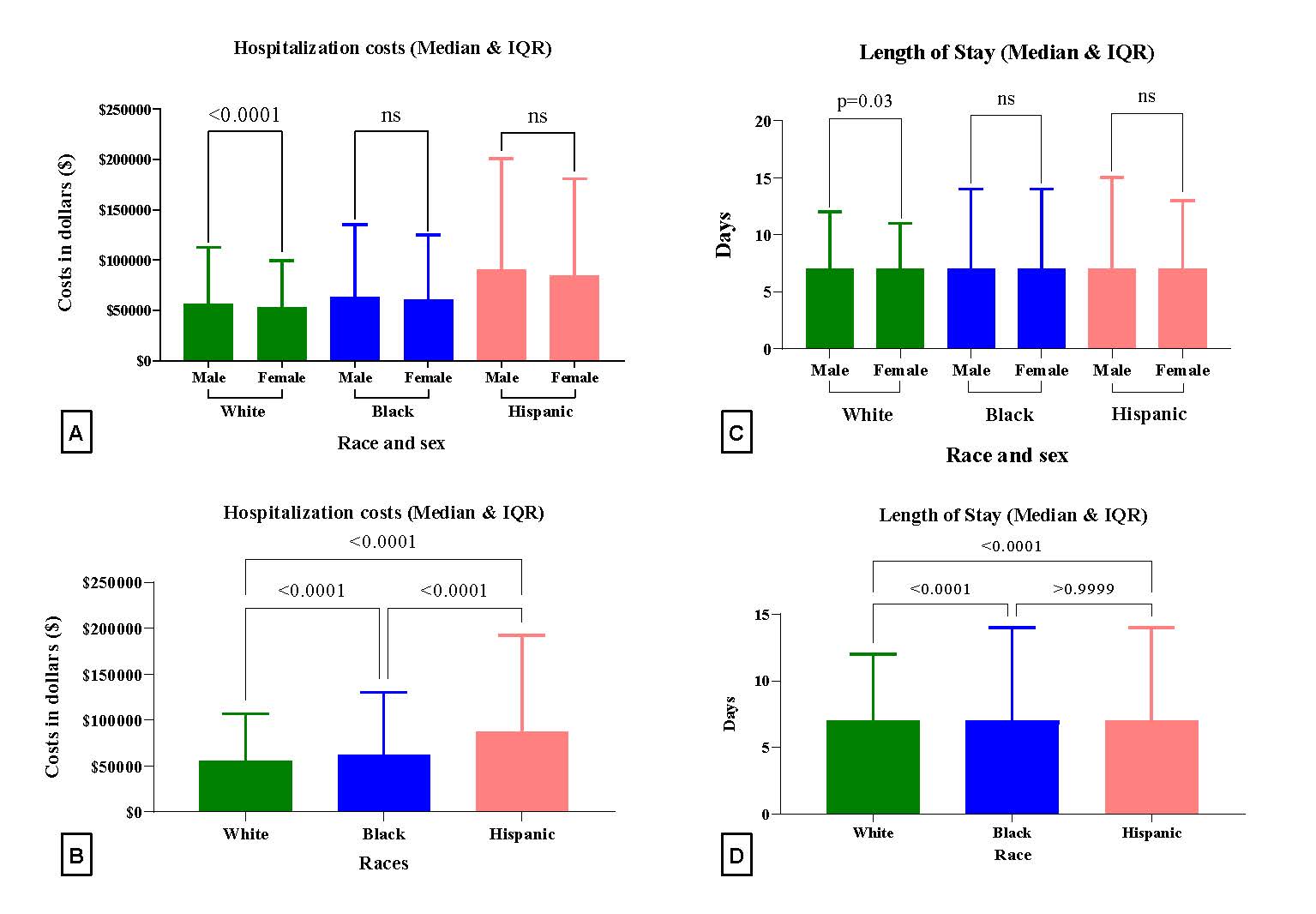
**

**Supplementary** **Figure 4:** Distribution of AHF with COVID-19 based on sex (1 and 2), and mortality rate per sex (3 and 4).


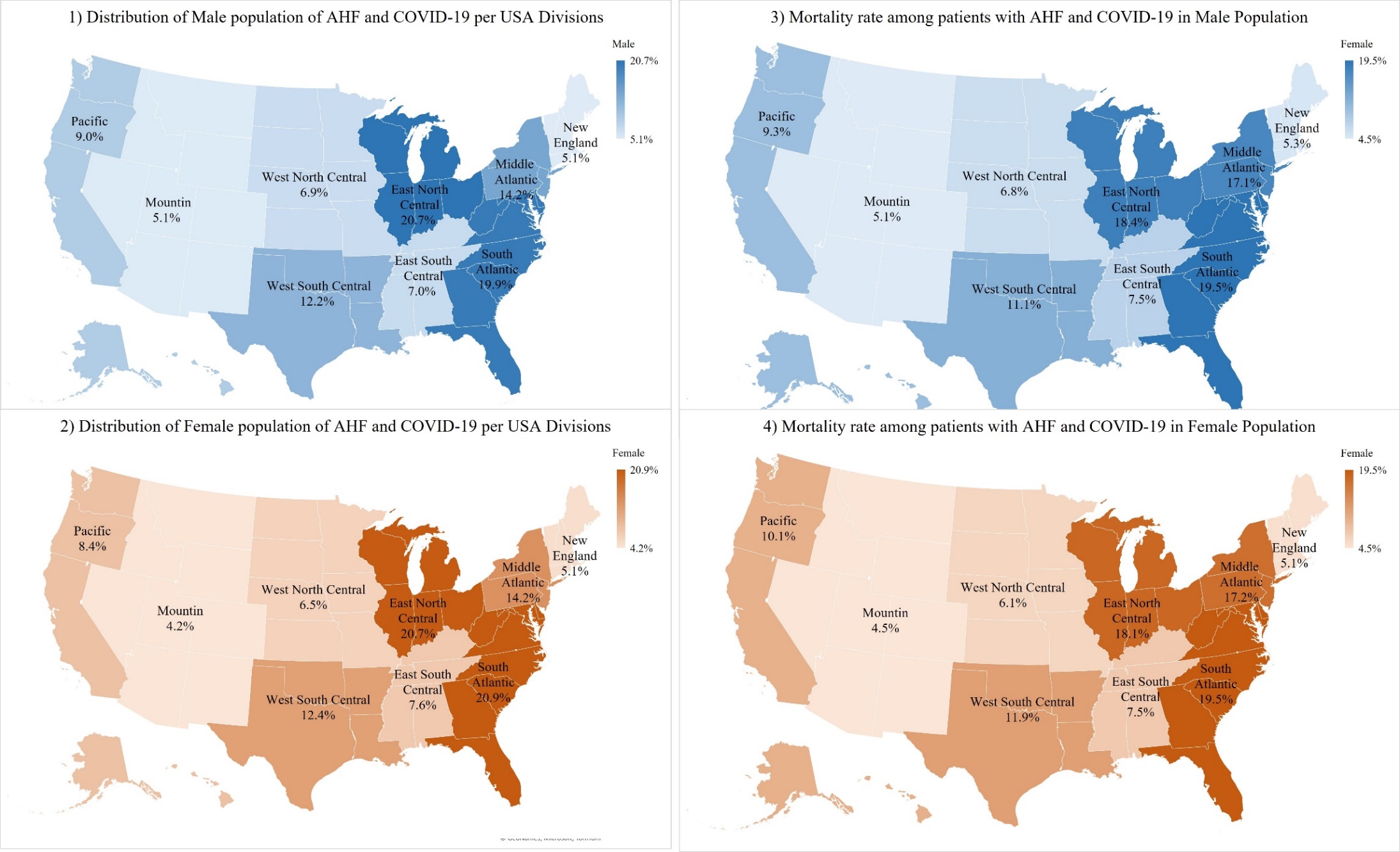

Supplement: Multimedia component 1 [file mmc1.docx]
